# A Legal Ban on Dog Meat Production: Political Decision-Making for an Ethical Community

**DOI:** 10.3390/ani14152269

**Published:** 2024-08-04

**Authors:** Yoojin Choi, Seola Joo, Myung-Sun Chun

**Affiliations:** Center for Animal Welfare Research, Research Institute for Veterinary Science, College of Veterinary Medicine, Seoul National University, Seoul 08826, Republic of Korea; ugenius1@snu.ac.kr (Y.C.); jsa321@snu.ac.kr (S.J.)

**Keywords:** dog meat, animal cruelty, animal ethics, public attitude, political turn

## Abstract

**Simple Summary:**

South Korea’s recent ban on dog meat production represents a significant political turn in the long-debated dog meat issue. To understand how this policy decision came about, it is necessary to examine how the Korean public’s perception of dog meat has changed from the past. This study’s findings show that the prevailing perception of consuming dog meat is negative in South Korea, largely due to concerns over animal cruelty. Nevertheless, oppositions remain against the ban from the view that it restricts the freedom to choose, which may delay improvements in animal welfare. However, the majority consensus to not tolerate unnecessary cruelty inflicted on dogs has been a powerful driver for politicians to legislate the ban on the dog meat industry. This study confirms that animal life is inherently political, urging us rethink and redefine the human–animal relationship.

**Abstract:**

For over 40 years, the human consumption of dog meat has been a controversial issue in South Korea. While some defend it as cultural tradition, others criticize the act as unethical treatment of dogs. This study examined the public perceptions and opinions driving the legislative efforts to ban dog meat through an online survey of 1000 participants assessing their experiences with dog meat, awareness of related issues, and support for institutional bans. The results revealed a widespread negative view of the dog meat industry, largely due to concerns over animal cruelty, and a negligible demand for consuming dog meat, which points to the expected demise of the industry. However, there was notable resistance to the ban from the view that it restricts personal freedom, with anthropocentric attitudes hindering progress in animal welfare. Regression analysis identified gender, political affiliations, animal experiences, and perceptions of dog meat as key predictors of support for the ban. As beloved pets, dogs influence human ethics, and the growing awareness of animal ethics has led to a ‘political turn’ enabling policymakers to enact bans. This study highlights how animal welfare benefits from political actions backed by societal consensus.

## 1. Introduction

In January 2024, the South Korean government passed the “Special Act on the End of Breeding, Slaughter, Distribution, and Sale of Dogs for Food Purposes”, banning the production and sale of dog meat for human consumption and effectively ending a highly contentious issue that had lasted nearly 40 years. However, the National Assembly’s decision to ban dog meat production came rather unexpectedly. Policymakers have made broad efforts to address the legal issues surrounding dog meat consumption from multiple angles since the 1980s, but these approaches have fallen short of resolving the controversies surrounding the consumption of dog meat. Especially in the past five years, a number of bills have been proposed to ban dog meat, only to be discarded, and the attempt to reconcile differing opinions through a Social Consensus Committee on the Dog Meat Issue, which was set up at the end of 2021, also failed to reach a decision after several rounds of discussions. Nonetheless, various political parties came together in December 2023 despite their differing views on other political issues and proposed a unified bill that finally led to the legislation of this law. Animal rights groups hailed it as a historic achievement and celebrated the end of the cruelty associated with dog meat production. However, dog meat producers expressed their displeasure and warned they would take further action to demand compensation for their loss of business. The media analyzed the motivation behind the law banning dog meat, pointing to the growth in the pet population and the larger pro-animal social climate in Korean society. However, viewing the law as a product of debates between conflicting parties or a clash between dog lovers and haters would be taking a narrow perspective. 

Korea has faced significant international criticism over its dog meat consumption policy, especially leading up to the 1988 Seoul Olympics. International organizations launched various campaigns, including protests, official complaints, and boycott threats. Such international criticism triggered negative reactions among Koreans and led to a more defensive stance during the 2002 Korea–Japan World Cup [1,2,3]. Some Koreans even began emphasizing the nutritional and economic value of dog meat [4,5,6]. Foreign scholars have interpreted this seeming advocacy of dog meat to be linked to the Korean identity and beliefs in its medicinal property [7,8], or the differences in attitudes towards dogs between East and West [1,9]. Others viewed it as local resistance to global trends [2], or as an outcome of conflict arising from differing perceptions of dogs among Koreans [10]. However, the conflict over dog meat consumption in Korea was not merely a response to international pressure or trends. The 1980s also marked a period of accelerated economic development in Korea, which lead to the greater production and accessibility of various meats for consumption. More Koreans also began keeping dogs as pets, leading to a pet boom in the 1990s. Legislation was also pursued to reflect the changing trends of society, and, in 1991, Korea adopted its first rudimentary Animal Protection Law that recognized the need for the humane treatment of animals in the society. 

As more people sided against the consumption of dog meat, a debate further ignited about the legal status of dogs [11] and the ethical dilemmas within the dog meat production process [12,13]. Ambiguities in the Korean law regarding the treatment of dogs, a prime example of how the variance in human attitudes towards animals across species, uses, and human benefits can lead to inconsistent regulations [14,15,16], have further complicated this discourse. In South Korea, dogs are protected as companion animals under the Animal Protection Act but are categorized as livestock under the Livestock Act. Additionally, dog meat production is not regulated by laws governing animal products or food hygiene, allowing it to operate outside the existing regulations on the slaughter and sale of animals for food purposes and sanitary supervision [17]. This legal gray area has allowed dog meat production to grow in scale [3] and also resulted in environmental and health issues, including consumer exposure to antibiotics, heavy metals, and drug residues [18,19]. The related government ministries have been criticized for using this legal ambiguity as an excuse for taking a passive stance and failing to do their jobs [17]. Ultimately, outdated laws and practices, combined with administrative neglect, have affected the lives of millions of dogs each year, posed risks to humans, and raised intense public criticism.

With the rising awareness and sensitivity about animal suffering, traditional practices involving animals are being challenged as public perceptions change, leading to efforts to regulate these practices in response to public opinion. For example, bullfighting, a long-standing national symbol of Spain, has been banned in parts of the country [20], and fox hunting was banned in the United Kingdom after more than 700 h of intense cross-party debate [21]. Taiwan [22] and Hong Kong [23] have banned the use of dogs for human consumption due to animal welfare concerns. Within the wide range of ethical perspectives on the use of animals from the animal rights position, which prohibits the use of animals, to the utilitarian animal welfare position, which permits it, these efforts demonstrate an approach to ethical issues regarding animals known as the ‘political turn’ in animal ethics. This ‘political turn’ suggests that animals can benefit from justice by establishing political structures that consider their interests, underscoring the need to address animal at the public level and securing the interests of animals through human duties [24]. Instead of applying a consistent set of principles to each issue or taking a purely moral or philosophical perspective, this approach proposes that pragmatically using a variety of concepts and methods to protect animal interests may be a practical alternative for advancing animal rights [25,26]. 

Implementing a ban on dog meat production in South Korea has been a highly contentious issue involving human norms around animal consumption and the government’s responsibility to protect animal interests in which public opinion has played a significant role in shaping policy decisions. As such, analyzing public attitudes toward banning dog meat in South Korea is crucial from the political-turn perspective. While previous surveys on the dog meat issue in South Korea have focused solely on gauging the agreement or disagreement with consuming dog meat, this study delves deeper into public attitudes and perceptions regarding dog meat consumption with the aim to provide a fuller picture of the current attitudes of the Korean public towards food and animals. The findings of this study provide insights into how the views of the Korean public have influenced political opinions and shaped policies aimed at protecting the interests of animals.

## 2. Materials and Methods

### 2.1. Questionaire

A survey was developed to gauge participants’ perceptions of dog meat consumption and their stance on banning dog meat production. The questionnaire consisted of five sections, referencing previous surveys conducted on dog meat in South Korea [27,28,29,30,31,32,33,34,35,36,37]. The first section inquired about participants’ legal knowledge concerning dog meat on a four-point Likert scale and their views on the pro–con arguments regarding the dog meat issue. The second section gathered data on participants’ experiences with dog meat consumption over the past decade, including reasons for consuming or not consuming it. Participants were also asked if they would consider consuming dog meat in the future and if they would recommend it to others. This section also explored positive and negative perceptions of dog meat at both individual and societal levels. The third section focused on whether the participants consented to legally banning dog meat production, captured on a four-point Likert scale. Participants were also asked to provide reasons for their agreement or disagreement with the dog meat ban. In the subsequent section, participants who supported the legal ban on dog meat production were asked to prioritize potential measures for its implementation in South Korea and to indicate their level of consent for increasing the government’s budget for banning dog meat. The last section of the questionnaire collected demographic information, including gender, age, educational level, household income, political orientation, religion, and pet ownership. Participants’ pro-animal attitudes were also measured using the Animal-Attitude Scale (AAS-10), a widely used tool for assessing individual differences in attitudes toward animal welfare issues and the human use of animals, with higher scores indicating a less permissive stance on the use of animals [38,39].

### 2.2. Data Collection and Analysis

After obtaining ethical approval from the Institutional Review Board of Seoul National University (IRB No. 2202/002-013), we conducted an online panel survey from 20–28 April 2022. Invitations to participate were sent to registered panel members. The survey employed quota sampling based on age (18 years and older), gender, and region, and a total of 1000 samples were collected and analyzed. It should be noted that the quota sampling recruited participants based on regional population ratios, which may not reflect the population distribution between urban and rural areas. However, despite this limitation, we believe the online panel survey is a useful tool for our purposes since the differences in online access between the regions in Korea is negligible. The survey results were summarized and compared between groups using Chi-square analysis, *t*-test, and ANOVA test. To predict participants’ level of agreement with legal ban of the production, sale, and purchase of dog meat, we used a stepwise regression model with independent variables such as demographic characteristics, animal experience and attitude, dog meat consumption, and perception. SPSS 26 was utilized for the statistical analysis.

## 3. Results

### 3.1. Demographic Characteristics and Dog Meat Consumption

In our survey, seventy-five percent of the participants attended or completed college, which is higher than the tertiary attainment of 25- to 34-year-olds (69%) and 25- to 64-year-olds (52%) reported in the 2021 OECD report [40]. Level of education is an important demographic characteristic to consider when interpreting the results, as education can influence the formation of attitudes. The average household income in South Korea in 2022 was 4,800,000 won (equivalent to 3840 US dollars), and almost half (46%) of the participants belonged to the above-average household income group. Half of the participants identified themselves as politically moderate, while 26.6% were progressive and 22.1% were conservative. In terms of religion, 54.5% of the participants were atheists, 13.6% followed Buddhism, and 31.9% practiced Christianity. Additionally, 40.5% of the participants reported owning a pet within the last ten years (Table 1). 

We found that 21.7% of the participants had consumed dog meat in the past ten years, with a lower percentage of women (10.3%) compared to men (33.2%, *p* < 0.001, χ^2^ = 76.90). The consumption of dog meat was higher among those who identified themselves as politically progressive (26.3%) and conservative (29.0%) compared to the moderate group (16.2%, *p* < 0.001, χ^2^ = 19.39). Furthermore, those who had consumed dog meat had weaker pro-animal attitudes according to the Animal-Attitude Scale-10 (29.73 ± 4.97, 33.90 ± 5.64, *p* < 0.001). On the other hand, 87.1% of the participants expressed that they do not intend to consume dog meat in the future. Among those who are willing to eat dog meat in the future, there was a higher percentage of males (21.1%) compared to females (4.8%) (*p* < 0.001, χ^2^ = 59.52). There was also a higher inclination to eat dog meat among those who identified as politically conservative (13.5%) and progressive (18.6%) compared to those who identified as moderate (10.1%) (*p* < 0.05, χ^2^ = 9.86). Additionally, individuals with experience in owning pets (8.1%) were less inclined to eat dog meat compared to those without such experience (16.1%) (*p* < 0.001, χ^2^ = 13.68). The Animal-Attitude Scale-10 scores of those who expressed willingness to eat dog meat in the future were also lower (27.87 ± 4.64 vs. 33.76 ± 5.52, *p* < 0.05) (Table 1).

The survey found that the primary reason for consuming dog meat (60.8%) was persuasion by others. Other reasons included considering dog meat a traditional seasonal food (30.4%), believing it to be healthy (18.4%), and enjoying its taste (18.0%). Among those who did not eat dog meat (78.7%), the main reasons were concerns about the cruelty associated with dog meat production (44.8%) and affection for dogs (33.7%). Another 25.5% expressed worries about unsafe production processes, while only 7.9% cited concerns about public criticism as a reason for not eating dog meat (Table 2). Among those who said they would eat dog meat in the future, 37.2% (*n* = 48) indicated a willingness to recommend it to others. Meanwhile, 53.9% (*n* = 470) of those who said they would not eat dog meat in the future expressed their intention to actively persuade others not to eat dog meat. However, more than half of the participants (51.9%) believed that dog meat consumption will continue in South Korea.

### 3.2. Perception of Eating Dog Meat

The debate surrounding dog meat consumption in South Korea has revolved around conflicting perspectives. Advocates emphasize the cultural and economic importance of dog meat, while opponents put forth arguments regarding the inhumane treatment of dogs and little value found in or held by the dog meat industry. Participants were asked to express their opinions on the controversial topic of dog meat production and consumption, which is frequently discussed in the media. 

The majority of participants agreed with the negative opinions on dog meat production and consumption. Specifically, 67.4% felt that dog meat is produced through animal abuse, while only 19% considered it a traditional cultural practice. Furthermore, 65.8% did not see the value of recognizing the dog meat industry as a part of the food industry, as opposed to 20.4% who did. Additionally, 63.2% viewed dog meat as harmful food, while 20.1% believed it is beneficial for human health. A significant 59.3% believed that pets should not be used for consumption, compared to 24.1% who saw no difference between pets and other livestock. Over half of the participants (54.6%) found the idea of eating dog meat repulsive, and about one-third respected individual preferences (Table 3). 

In addition to these results, 79.4% of participants had negative views towards dog meat consumption, and 93.4% felt that society holds a negative view of it as well. Despite historical context, the support for dog meat consumption showed a clear decline (Table 4).

### 3.3. Awareness of Dogmeat Issue and Political Expression in Legal Intervention

Participants were asked to rate their awareness of important legal issues related to dog meat production on a 4-point scale. A significant number of participants (55.3%) were completely unaware that dogs are considered livestock and can be mass-bred under the Animal Husbandry Act. Similarly, 44.0% were uninformed about the lack of legal regulations on slaughtering dogs, and 37.1% were unaware that dogs are not legally defined as food. About 40% had some level of awareness that the Supreme Court had deemed the electrocution of dogs illegal (28.0% to some extent and 8.7% to a great extent) and that there is a Social Consensus Committee working to eradicate dog meat (31.0% to some extent and 6.0% to a great extent) (Table 5).

The survey results on participants’ consent to a legal ban on dog meat showed that approximately 64% of the participants supported legal intervention to ban the consumption of dog meat. They believed that such a prohibition reflected the humanity of our society (61.5%) and aligned with international standards for the humane treatment of animals (51.0%). Over 40% also believed that humane dog meat production is not feasible. However, around 36% of the participants did not agree with legally banning dog meat and overwhelmingly selected the statement that people have the right to eat whatever they want (81.3%) (Table 6). 

In addition, those who supported legal intervention prioritized using legislation to prohibit dog meat consumption (97.8%, *n* = 627), followed by the need for a shift in public perception (97.3%, *n* = 624), and strengthening penalties for illegal producers (96.3%, *n* = 617). They also emphasized the importance of rescuing dogs from dog farms (94.5%, *n* = 606) and implementing measures against dog meat producers (88.8%, *n* = 569). We found that 46.7% of participants believed that the legal prohibition of dog meat consumption in South Korea will succeed within the next ten years. However, 18.1% of participants expressed pessimistic views, stating that it will never be fully implemented. 

### 3.4. Regression Analysis

We assessed three models for predicting consent to ban dog meat using hierarchical regression analysis to determine the effect of the predictors. The first model included individual characteristics such as gender, age, education, income, political orientation, and religion. The second model included predictors related to animal experience, such as pet ownership and pro-animal attitude. The third model comprised predictors pertaining to perceptions about dog meat, including an understanding of dog meat issues, experience with consuming dog meat in the past ten years, and negative perceptions of dog meat consumption.

In the first model considering individual characteristics (R^2^ = 0.068, F = 8.035, *p* < 0.001), gender was found to be the most significant predictor, with a beta value of 0.23 and a *p*-value of less than 0.001, explaining 6.8% of the variance. Additionally, conservative political orientation showed a negative correlation (β = −0.089, *p* < 0.01). When animal experience was added in model 2, the variance explained increased significantly (R^2^ = 0.339, F = 46.148, *p* < 0.001, ΔR^2^ = 0.271). Age (β = 0.111, *p* < 0.001), conservative political orientation (β = −0.058, *p* < 0.05), pet ownership (β = 0.124, *p* < 0.001), and pro-animal attitude (β = 0.524, *p* < 0.001) were identified as significant predictors in model 2. The third model, which further incorporated dog meat perception predictors, explained an additional 21.2.% of the variance (R^2^ = 0.551, F = 80.542, *p* < 0.001, ΔR^2^ = 0.212). In model 3, age (β = 0.085, *p* < 0.001), animal experience (pet ownership, β = 0.069, *p* < 0.01; pro-animal attitude, β = 0.242, *p* < 0.001), and the four added variables representing the dog meat experience and perception were found to be significant. Notably, pro-animal attitude (measured by AAS-10) emerged as a significantly correlated predictor of consent to ban dog meat in both model 2 and model 3. Moreover, participants’ negative perception of dog meat consumption was identified as the strongest predictor of support for banning dog meat (Table 7).

## 4. Discussion

### 4.1. Perception Shift of Dogs from Meat Source to Companion Animals

This study discovered that the public opinion on consuming dog meat in South Korea is not highly polarized. The majority of participants, approximately 80%, stated that they had not consumed dog meat in the past ten years. The result is a complete reversal from a survey conducted 25 years ago, in which 83% of participants said they had eaten dog meat [4]. Furthermore, 87.1% indicated that they have no intention of eating dog meat in the future, with half of these participants expressing that they would actively encourage others to refrain from consuming it. Thus, dog meat consumption is expected to gradually decrease, ultimately leading to a decline in the dog meat industry. Additionally, dog meat was widely considered inappropriate as food. Among those who had not consumed dog meat in the last decade (78.3%), reasons cited for deeming it inappropriate as food included concerns about cruelty (44.8%, *n* = 351) and affection for dogs (33.7%, *n* = 264). Some also expressed worries about the health risks associated with dog meat production (25.5%, *n* = 200), while others felt there was simply no reason to eat it (28.6%, *n* = 224). Approximately 20% of participants reported consuming dog meat within the last ten years, with a small number still eating it as a seasonal food (30.4%, *n* = 66) or for physical fitness reasons (18.4%, *n* = 40), diverging from past justifications for consuming dog meat, such as improving physical strength and sexual performance [41] or improving the health of the elderly [42]. It was found that men with weak pro-animal attitudes and those with more visible political leanings were most likely to continue consuming dog meat. This is consistent with a study from Vietnam, which found that consuming dog meat was linked to masculinity, and consuming brutally produced dog meat together was encouraged to create a distinct culture and foster intimacy [43].

The consumption of dog meat has long been seen as a source of sharp conflict in South Korea; however, the findings of this study revealed that public perception of the issue is not heavily divided today. The majority of people believe that the dog meat industry is based on cruelty (67.4%) and is unworthy of protection (65.8%). Supporting opinions, such as viewing it as a traditional culture (19.0%) or a legitimate industry (20.4%), are in the minority. Furthermore, the nutritional value of dog meat (20.1%) is considered to be of little value, with 63.2% of people perceiving it as harmful and unhygienic food. In other words, most Koreans do not see dog meat as part of their traditional culture but rather as illegal animal cruelty. The prevailing opinion is that dogs should not be consumed because they are considered as pets (59.3%), outweighing the utilitarian perception that they are no different from other domesticated animals (24.1%). Previously, some politicians had suggested that recognizing the dog meat industry as a legitimate livestock sector could help resolve disputes, but this idea was rejected and even met with backlash [1]. This reflects the cultural sentiment in Korea that views dogs as companion animals rather than livestock. Additionally, negative perceptions of dog meat are prevalent at the societal level (93.4%). In other words, the current atmosphere around the issue is such that it lends a stronger and more active voice to those who have decided not to consume dog meat. This suggests that there is already strong social pressure on dog meat advocates and the dog meat industry in South Korea.

This study also found that owning a pet did not necessarily influence dog meat consumption in the past, but pet owners were less likely to eat dog meat in the future (past 18.8% vs. future 8.1%), which suggests that pet ownership could change attitudes towards dog meat consumption. This finding goes against earlier studies that suggested Koreans make a distinction between edible dogs and companion dogs [9,44], or that their attitudes towards dog meat are deeply rooted and unrelated to pet ownership [7]. A survey on dog meat consumption in China also corroborates the idea that pet ownership can influence attitudes towards dog meat [45]. Meanwhile, age did not have an impact on future dog meat consumption, which diverges from previous studies that suggested younger people would be more likely to oppose dog meat consumption and eat less [2,8]. In South Korea, families with more than three members are the primary owners of pet dogs (26.9% of families with three members, and 29.3% of families with more than four members) [46], which generally comprise members spanning across at least two generations. The relationships we form with our companion animals involve a deep and ongoing personal connection that goes beyond mere caregiving [25,47]. When caring for pets, humans often feel a sense of ethical responsibility toward them. In this sense, in South Korea, differences in attitudes towards animals between generations may be less significant within a family setting.

Amid the growing recognition that our choices around food are tied to ethical considerations [48], Koreans have become more aware of the connection between the production of dog meat and the suffering of dogs. Animal protection organizations and the media have played a crucial role in raising public awareness about animal abuse and ethical issues [49], evoking strong emotions by appealing to the public about issues of animal cruelty [26]. This study showed that consuming dog meat in South Korea is no longer a controversial issue, as there was already a widespread ethical consensus to end the cruelty associated with dog meat, and this had effectively become a social norm. Thus, it can be said that the legislation of dog meat banning is not a forceful enforcement to restrain a traditional practice but represents a political turn of Korean society’s ethical consensus.

### 4.2. Complex Attitudes toward Institutional Intervention for Banning Dog Meat Production

Legal measures have the power to shape public opinion on dog meat consumption [45,50]. When personal ethics are insufficient to prevent animal abuse, society often relies on the legal system for assistance [51]. Asdal and Druglitrø argue that legislation to protect animals from cruelty or ensure their welfare reflects how society organizes itself, and the human–animal relationship ultimately pertains to human issues [52]. This study found that 64.1% of participants supported legal intervention to ban dog meat, citing that it showcased the compassion of Korean society (61.5%, *n* = 394) and was an effective way to stop the inhumane treatment of animals (51.0%, *n* = 327). More than half (51.0%, *n* = 327) also endorsed international trends focusing on animal welfare, believing that ensuring the well-being of dogs is intrinsically good.

Nonetheless, translating ethical concerns for animals into legal measures requires acknowledging the impossibility of universal agreement [51], and, in this study as well, it was found that people hold complex perspectives on using laws to regulate human conduct. While the intention to consume dog meat is low (12.9%), there is relatively high resistance (35.9%) to legal intervention in dog meat production. It is important to note that this resistance does not indicate a willingness to eat dog meat or condone animal cruelty, since more than half of all participants (54.6%) found eating dog meat repulsive. However, a sizable portion (31.8%) considered it a matter of personal preference, and, among those opposed to regulating dog meat consumption, the majority (81.3%, *n* = 292) believed that individuals should have the right to freely choose what they eat. That is, many valued and respected the right to make personal dietary choices, and the right to eat what they want is seen as a significant benefit of opposing legal intervention. For these people, legal intervention in dog meat production elicits a strong negative reaction, regardless of whether they intend to actually consume dog meat. The conflicting attitude stemming from this desire to protect their right to choose from legal interference has been one of the sources of discord in reaching a consensus on banning dog meat production. 

Furthermore, personal characteristics and experiences significantly influenced attitudes toward legal intervention, highlighting the relevance of this research at the individual level. Regression analyses revealed that support for institutional interventions was significantly associated with gender, age, political affiliation, animal experience, and perception of dog meat. The data indicated that women were more supportive of a dog meat ban, confirming gender differences in attitudes toward animal-related issues found in previous studies [20,39,53,54]. Gender differences are also related to attitudes towards animals, with men often affirming their masculinity through animals, while women are more inclined to care for animals [54,55]. Our results predicted that men with weaker pro-animal attitudes or a stronger political orientation tend to not only have stronger intentions to eat dog meat but also be more opposed to institutional intervention. Negative perceptions of dog meat were a particularly strong predictor of support for intervention: those who had not eaten dog meat in the past and those with a high awareness of the systemic problems associated with dog meat were predicted to have negative opinions and were found to be more likely to support intervention.

Overall, participants were not well-informed about how the present system places dogs in conflicting situations (see Table 5). Because the awareness of dog meat production is correlated with the level of support for a legal ban on dog meat production, raising awareness about the cruel treatment of dogs in the dog meat production process could gather more support for legal interventions. A precise and wider recognition of how the same animal is treated unequally across legal frameworks can elevate public demands for institutional changes and create opportunities to align the legislation with higher welfare standards [14,15].

### 4.3. Political Decision Protecting Animals’ Interests

The law reflects a society’s moral and ethical beliefs. In a democratic system, elected officials rely on the political process of majority rule to balance interests [51]. However, including animal interests as part of the community’s interests is challenging, as animal interests often conflict with human interests [56]. Creating laws for animals is a human issue [52], and policymakers need to determine what benefits the public will gain and what human interests can be sacrificed for the sake of animals, and whose interests will be compromised. Generally, animal exploitation is often institutionalized and perceived as ‘necessary suffering’ rather than abuse when substantial economic benefits for humans are anticipated [15]. However, regarding dog meat production, this study clearly found that the Korean public no longer perceives the suffering endured by dogs for human benefits as necessary suffering (96.3%, *n* = 617). In other words, the public’s ethical stance on tolerating cruelty towards animals has surpassed the previous tolerance threshold. The lack of internal inconsistency [14,15] between Korea’s strong animal protection laws and the institutional environment that allows dog meat consumption may have further provided the public with a reason for a legal ban.

The Social Consensus Committee, including members of relevant government agencies, animal protection organizations, and dog meat producers, had held over twenty discussions in 2022 to bring out a social consensus on dog meat consumption but failed to reach a conclusion. However, the law banning dog meat production was passed with overwhelming support from the members of Korea’s National Assembly, thereby effectively recognizing dogs as companion animals rather than livestock. Politicians tend to prioritize the interests of the voters who elected them over the welfare of animals [56], and the legislation of this ban shows that, for Korean politicians, success in banning dog meat production represents the majority’s opinion and does not harm their interests. The decision to put a stop to the entrenched freedom to consume dog meat and forgo the economic benefits from traditional but illegal production could be justified by the negative public perception of dog meat. That is, the societal sentiment that views the cessation of dog meat consumption to be aligned with the interests of the majority surpassed the threshold for policy decisions and became the driving force for institutional reform.

The “Special Act on the End of Breeding, Slaughter, Distribution, and Sale of Dogs for Food Purposes” enacted by the National Assembly was announced as an imperative task to propel the nation towards becoming a leading country in animal welfare, and as a decision that aligns with the current demands of the times [57]. By banning the breeding, slaughtering, and distribution processes of dogs for meat, the law compromises the interests of those involved in producing dog meat and those who want to eat dog meat. A legal ban does not necessarily lead to the complete cessation of the practice [58], and ongoing resistance from those adversely affected by the ban is expected. Supporters of the ban overwhelmingly expressed that improving the treatment of dogs alone was insufficient; there must also be support and measures for those who suffer losses due to the ban (88.8%, *n* = 569). Reflecting this, policymakers have implemented feasible measures such as a three-year grace period for business closure and consulting support for transitioning to other jobs [59]. Tracking the progress of these legal measures and future steps, along with discussions about their implementation from the perspectives of stakeholders, will help in understanding the post-ban landscape.

Korea’s legal ban on dog meat, viewed in relation to the findings of this study, is a case where societal consensus played a role as a political pressure, even though not all members of society share the same ethical position on animal use [60,61]. Although this study focuses specifically on the unique context of dog meat consumption in Korea, and thus does not address issues related to other animals, it highlights the broader implications of the dynamics of the political turn in animal ethics. The public’s experience in how strengthened animal welfare standards and shifting attitudes towards animals can determine the fate of an industry is likely to serve as a catalyst in fostering increased public attention to other animal welfare issues. 

## 5. Conclusions

The issue of dog meat consumption in South Korea has been a hotly debated topic for over 40 years. Concerns about unnecessary animal cruelty in the process of dog meat production have evolved the issue into an ethical and relational matter, posing a challenge for policymakers to embrace a “political turn in animal ethics” [24]. In this context, this study aimed to understand how Koreans’ perceptions of dog meat have changed today and the underlying public opinions driving legislative intervention. The findings revealed that the majority of the public viewed dog meat consumption negatively, largely due to the perceived suffering inflicted on dogs. However, despite the decreasing demand for dog meat, there was still significant resistance to legal regulations that could limit individual freedom. This human-centric attitude has been one of the key obstacles to resolving animal welfare issues. 

While public attitudes toward animals may be inconsistent, dogs, as representative companion animals, have demonstrated the potential to influence human ethical attitudes through their intertwined lives. Dogs have gained political support from animal protection organizations, the media, researchers, and the court, and the public awareness of cruelty in dog meat production has prompted political action. The ban on dog meat production in South Korea, implemented in January 2024, was a politically motivated decision driven by long-standing public ethical concerns about animals’ interests that carefully considered the interests of dogs and ensured a consensus aligning with the voter majority. Understanding this decision as a political one affirms that animal lives are inherently political, demanding us to rethink human–animal relationships and redefine our political community in a way that could enable us to make more just decisions for both humans and animals.

## Figures and Tables

**Table 1:** Demographics of participants.

Groups	Sub-Groups	*n* (1000)	Have Eaten Dog Meat for the Last 10 Years		Will Eat Dog Meat in the Future	
			*n* (% in the Group)	*p* Value (χ^2^)	*n* (% in the Group)	*p* Value (χ^2^)
Gender	Men	497	165 (33.2)	0.000 (χ^2^ = 76.90)	105 (21.1)	0.000 (χ^2^ = 59.52)
	Women	503	52 (10.3)		24 (4.8)	
Age	18–29	171	31 (18.1)	0.213 (χ^2^ = 5.82)	18 (10.5)	0.837 (χ^2^ = 1.442)
	30–39	150	29 (19.3)		18 (12.0)	
	40–49	187	37 (19.8)		25 (13.4)	
	50–59	195	42 (21.5)		26 (13.3)	
	Over 60	297	78 (26.3)		42 (14.1)	
Education	Completed middle or high school	242	61 (25.2)	0.129 (χ^2^ = 2.05)	36 (14.9)	0.292 (χ^2^ = 1.11))
	Attended or completed college	758	156 (20.6)		93 (12.3)	
House income monthly (10,000 Korean won/8 dollar)	Under 200	127	27 (21.3)	0.260 (χ^2^ = 4.01)	15 (11.8)	0.407 (χ^2^ = 2.90)
	200~500	431	89 (20.6)		55 (12.8)	
	500~700	223	43 (19.3)		24 (10.8)	
	Over 700	219	58 (26.5)		35 (16.0)	
Political orientation	Progressive	266	70 (26.3)	0.000 (χ^2^ = 19.39)	36 (13.5)	0.007 (χ^2^ = 9.86)
	Moderate	513	83 (16.2)		52 (10.1)	
	Conservative	221	64 (29.0)		41 (18.6)	
Religion	Buddhism	136	25 (18.4)	0.387 (χ^2^ = 3.03)	13 (9.6)	0.246 (χ^2^ = 4.19)
	Protestantism	210	51 (24.3)		34 (16.2)	
	Catholicism	109	28 (25.7)		11 (10.1)	
	Other/No religion	545	113 (20.7)		71 (13.0)	
Pet owning (last 10 years)	Pet owner	405	76 (18.8)	0.063 (χ^2^ = 3.45)	33 (8.1)	0.000 (χ^2^ = 13.68)
	Not pet owner	595	141 (23.7)		96 (16.1)	

**Table 2:** Reasons for (not) eating dog meat (*n* = 1000, multiple choice).

Dog Meat Consumption	Reason	*n* (%)
I have eaten dog meat (*n* = 217)	because people encouraged me to eat it.	132 (60.8)
	because it is a customary seasonal food.	66 (30.4)
	because it is good for health.	40 (18.4)
	because it tastes good.	39 (18.0)
	because there was no particular reason not to eat it.	18 (8.3)
I have not eaten dog meat (*n* = 783)	because the way dogs are raised and slaughtered for food is cruel.	351 (44.8)
	because I like dogs.	264 (33.7)
	because there is no particular reason to eat it.	224 (28.6)
	because dog meat is not safely produced.	200 (25.5)
	because there is no one around me who eats it.	125 (16.0)
	because it does not taste good.	84 (10.7)
	because the public criticizes eating it.	62 (7.9)

**Table 3:** Pro and con arguments about dog meat (%, *n* = 1000, multiple choice).

Arguments	Pros	Neutral	Cons	Arguments
Eating dogs is a part of traditional culture.	19.0.	13.6	67.4	The process of producing dog meat results in animal cruelty.
Dog meat production should be recognized as a normal food industry.	20.4	13.8	65.8	Dog meat production is not important nor valuable in food industry.
Dog meat has nutritional value.	20.1	16.7	63.2	Dog meat is not safely produced under the current legal system.
Eating dogs is no different from eating other animals.	24.1	16.6	59.3	Dogs are companion animals that humans should not be allowed to eat.
Eating dogs is a personal preference.	31.8	13.6	54.6	Eating dogs is considered disgusting in public sentiment.

**Table 4:** Perception of eating dog meat (%, *n* = 1000).

Perception	Very Positive	Positive	Negative	Very Negative
Public perception of dog meat	0.4	6.2	65.3	28.1
My perception of dog meat	1.9	18.5	59.2	20.4

**Table 5:** Awareness of legal and political issues regarding dog meat in South Korea (%, *n* = 1000).

Statement	Degree of Awareness			
	Not at All	Very Little	Somewhat	To a Great Extent
Under the Livestock Industry Act, dogs can be legally raised as livestock.	18.9	55.3	23.0	2.8
Slaughtering dogs are not legally controlled under the Livestock Products Sanitary Control Act.	13.9	44.0	35.6	6.5
Dog meat is not legally defined as food under the Food Sanitary Act.	14.2	37.1	39.6	9.1
The Supreme Court ruled that killing dogs with electricity violates the Animal Protection Act.	17.0	46.3	28.0	8.7
In 2021, the Social Consensus Committee was established to ban dog meat production, sale, and purchase.	17.1	45.9	31.0	6.0

**Table 6:** Consent to a legal ban on dog meat (%, *n* = 1000, multiple choice).

Statement	Reason (Multiple-Choice)	Percentage (%)
I (strongly) consent to a legal ban on using dogs as food (*n* = 641)	because banning the production, sale, and purchase of dog meat reflects the humanity of our society.	61.5
	because caring for sentient animals is an international standard.	51.0
	because the humane production of dog meat is impossible.	42.7
	because securing one’s interest in harming other animals should not be legally allowed.	34.3
I (strongly) do not consent to a legal ban on using dogs as food (*n* = 359)	because people have the right to eat what they favor.	81.3
	because it is impossible to ban someone from pursuing their interest.	35.7
	because protecting people in dog meat production is important.	25.6
	because humans have priority over animals.	16.2

**Table 7:** Hierarchical regression analysis predicting the consent to dog meat bans.

	Model 1 (Individual Characteristics)			Model 2 (Animal Experience)			Model 3 (Dog Meat Perception)		
	B	SE	β	B	SE	β	B	SE	β
(Constant)	2.437	0.17		−0.131	0.214		−1.069	0.202	
Gender (Women)	0.421	0.057	0.23 ***	0.072	0.052	0.039	0.009	0.043	0.005
Age group	0.013	0.02	0.02	0.07	0.017	0.111 ***	0.053	0.014	0.085 ***
Education	0.051	0.033	0.05	0.046	0.028	0.045	0.029	0.023	0.029
House income	−0.009	0.03	−0.009	−0.013	0.025	−0.014	−0.023	0.021	−0.025
Political orientation (Progressive)	0.045	0.068	0.022	−0.074	0.058	−0.036	−0.001	0.048	0.001
Political orientation (Conservative)	−0.197	0.074	−0.089 **	−0.127	0.062	−0.058 *	−0.084	0.052	−0.038
Religion (Buddhism)	0.147	0.088	0.055	0.027	0.074	0.01	−0.009	0.062	−0.003
Religion (Protestantism)	0.049	0.073	0.022	0.104	0.062	0.046	0.062	0.051	0.028
Religion (Catholic)	−0.034	0.095	−0.012	−0.039	0.08	−0.013	−0.045	0.066	−0.015
Pro-animal attitude (AAS-10)				0.083	0.005	0.524 ***	0.038	0.004	0.242 ***
Pet ownership (Yes)				0.23	0.05	0.124 ***	0.129	0.042	0.069 **
Awareness of dog meat issue							0.017	0.007	0.058 *
Dog meat in last 10 years (No)							0.131	0.054	0.059 *
Negative perception (Society)							0.088	0.043	0.054 *
Negative perception (Personal)							0.653	0.04	0.487 ***
R^2^	0.068			0.339			0.551		
Adjusted R^2^	0.060			0.332			0.544		
ΔR^2^				0.271			0.212		
F	8.035 ***			46.148 ***			80.542 ***		

* *p* < 0.05, ** *p* < 0.01, *** *p* < 0.001.

## Data Availability

All data and results related to this study are original and included in the article.
